# Notes and News

**Published:** 1887-06-25

**Authors:** 


### Notes and News.

COLLECTED AND COMMUNICATED.

DR. HENRY FITZGIBBON, who is a brother of Lord Justice Fitzgibbon, has just been elected to the vice-presidency of the Irish College of Surgeons, and will accordingly become president next year.

MISS HANNAH TURNER, Sister of Martha Ward, St. Bartholomew's Hospital, has been appointed Lady Superintendent to the Children's Hospital, Pendlebury, near Manchester. Miss Turner gained the Gold Medal for proficiency in nursing, awarded half-yearly at St. Bartholomew's, in October 1885.

DR. WILLIAM RAMSEY has been appointed Professor of Chemistry in University College, London, vacant by the resignation of Dr. Williamson. Dr. Sydney Ringer, F.R.S., has been appointed Holme Professor of Clinical Medicine, in succession to the late Dr. Wilson Fox. Mr. Victor Horsley, F.R.S., has been appointed Professor of Pathology, in succession to Dr. Bastion, resigned. The first award of the Slade Travelling Scholarship (value £150 per annum for two years) has resulted in the election of Mr. Harrington Mann, of Glasgow.

AT Lincoln the Hospital Saturday collection is to be helped by a somewhat extensive bazaar. Neighbouring towns and villages are asked to contribute their share, and many of them are having stalls of their own. Gainsborough, Sleaford, and other places are well to the front, but Horncastle seems to be keeping as well to the rear. Why is this? Has the vicar or other leading citizen no interest in the Hospital Saturday collection? or is it merely an accidental circumstance? Perhaps it is because what is everybody's business is nobody's business; but, at any rate, it will be discreditable to Horncastle if steps are not taken to secure that its proportion of needful help shall be forthcoming. The secretary of the committee said that he had twice written to Horncastle on the subject, but had received no reply, and he was not disposed to go hat in hand to the executive of that town. Horncastle must look both to its credit and its manners.

IN view of the Hospital Sunday Fund this year being deprived of the annual collection at Westminster Abbey to-morrow, it was arranged that one-third of the collection at the second Jubilee Service on Wednesday should be devoted to the Fund. A third will be given to Westminster Hospital, and the remaining third is to be divided between the Hospital Saturday Fund and the Western Dispensary.

QUARTER-MASTER NEWTON H. NIXON, Volunteer Medical Staff Corps, has obtained permission from the National Rifle Association to put collecting-boxes again this year in Wimbledon Camp during the meetings in July, in aid of the London hospitals, and it is hoped that Volunteers and the general public will respond liberally.

THE sisters at the North Ormsby Cottage Hospital have been able to secure money, eggs, milk, rhubarb, flowers, and newspapers, as contributions to their institution, but they do not seem able to obtain any garden-seats. Will not somebody in Ironopolis, which is so very near to North Ormsby, send them a score or two of these very comfortable adjuncts to Hospital treatment?

THE Hartlepools' Hospital committee have done wisely to rescind a very unsatisfactory resolution which had been passed at the previous meeting. That resolution permitted members of the committee of management to tender for the supply of goods to the hospital, a practice which should on no account be encouraged. The good-sense of the latter meeting has, however, decided to prevent any such unseemly occurrence.

AT Newcastle-on-Tyne, as at other places, the Hospital Sunday Fund has this year been considerably augmented, a total of £4,073 remaining for distribution after the payment of all expenses, the increase being not less than £500, or one-eighth more than the amount collected last year.

FRONTING one side of that little oasis at Paddington known as the "Green," stands a building, familiar to most of us—the Paddington Children's Hospital. Externally its unfinished state shows the pinch of poverty and the hard struggle which the institution has had for existence. The hospital is now four years old, and already it has a noble record of work. Last year 10,624 new out-patients were received, while the in-patients numbered 309. Two kind friends endowed a couple of "In Memoriam" cots. We are glad to see that the committee are turning their attention to the out-patient accommodation, for at present the over-crowding is exceedingly bad for the little sufferers who are brought hither. At the rear of the hospital the committee have purchased an excellent freehold site for £2,587, and on this it is proposed, when the funds can be collected, to build a new out-patients' wing. A sum of £2,000 is required for this purpose, and we earnestly hope the friends of this institution will heartily assist in the raising of it.

THE following vacancies are announced this week, the amount of the salary being placed in each case after the name of the institution :—

**Honorary Medical Officers.**

Westminster Dispensary. Honorary Surgeon.

**Resident Medical Officers.**

Coventry Hospital. House Surgeon. Doubly-qualified.—£100.
Hospital for Sick Children, Great Ormond Street. Assistant Physician.
St. Luke's Hospital. Clinical Assistant. Doubly-qualified.
Westminster Hospital. Surgical Registrar.—£40.

**Non-resident Medical Officers.**

National Dental Hospital. Lecturer on Operative Dental Surgery.
Ditto ditto. Anæsthetist.
Western Ophthalmic Hospital. Dispenser. Qualified.

**Trained Nurses.**

Bodmin Asylum. Head.—£35.
Bradford, New Children's Hospital. Charge Nurse.—£20.
Burnley Victoria Hospital. Head.—£26.
London Temperance Hospital. Night.—£25.
Margate Infirmary. Head.—£30.
Middlesbrough Fever Hospital. Charge.—£30.
Peterborough Nursing Association. One.—£30.
West Bromwich Hospital. One.—£20.
West London Hospital. Assistant.—£24.

---

THE Poplar Hospital for Accidents is a very popular institution, and deservedly so. It has just held its annual festival, under the presidency of Mr. Sydney Buxton, M.P. Mr. Buxton indicated why the hospital had secured the good opinion of all men. It was situated exactly where its services were most needed. It was admirably officered by its efficient medical and surgical staff—some of whom, it is significant to note, are what are called "General Practitioners." It was managed thoroughly, efficiently, and economically by its diligent committee and its excellent secretary ; and to crown all, it was out of debt, had a balance at the bank, and had recently purchased the freehold. Bravo Poplar ! Long may it continue to be a hardworking and prosperous institution.

---

THE annual meeting of the Donors and Governors of the Wigan Infirmary has been held. The total receipts for the year amounted to £5,129 13*s.* 6*d.*, and the expenditure to £4,777 9*s.* 4*d.* The report adds, "Warmest thanks are heartily tendered to the contributors on Hospital Sunday, and to the working-classes for their continued and handsome liberality in connection with the weekly and Hospital Saturday contributions. To their honour be it said, that during the last year no less than the sum of £2,759 16*s.* has been paid over to the institution."

---

THE City Orthopædic Hospital in Hatton Garden is not just at present in the happiest of conditions. Though it has treated no less than 52,000 cases of deformity in a space of thirty-six years, it is now urgently in need of pecuniary help. Year by year the applicants for admission increase in numbers, though only some twenty cases at a time can be received. The out-patients, too, crowd the waiting-room, and many have to return to their homes unseen, because the staff is totally insufficient to deal with the cases. The lease of the hospital is expiring, and cannot, we understand, be renewed. The committee have set apart a nucleus of £2,000 for a new building, and await the timely assistance of the benevolent to help them.

A HOSPITAL SECRETARY writes to us :—"I do not know whether the Council of the Hospital Sunday Fund, among the papers which they send to clergymen and ministers this year, include your capital Special Number ; but if not, I should be greatly obliged if you would send me a couple of copies, which I will bring under the notice of two leading preachers in my neighbourhood. In former years I have taken a similar step, but in drawing the attention of these gentlemen to the real position of the London medical charities (of which, despite the papers of the Fund, they seemed to be practically unaware), I had no such powerful "brief" as that which your Special Number puts in my hands. I venture to think that a still more condensed statement is what is really needed for this particular purpose."

---

AT Worthing, Hospital Sunday has become a recognised and much appreciated institution. It is said that the local infirmary has already largely benefited by it, and an effort is to be made this year to still more augment its usefulness. All the churches and chapels, with but one exception, will have a hospital collection on the second Sunday in August.

---

AT Pudsey, near Leeds, a Cottage Hospital is in contemplation, and it is proposed that a recreation ground shall also be provided for the town. There seems to be some little difference of local opinion, but all manufacturing centres like Pudsey should be adequately provided with hospital accommodation, and also with large open spaces for the recreation of the public, and especially of children. We hope the local leaders will sink their disagreements, and unite heartily for such excellent objects as those proposed.

---

IT seems that it must be true that we are rapidly becoming "hospitalised," for the latest movement afloat is one for the establishment of a hospital, not for the treatment of the diseases of the little finger or the great toe, but for the study of diseases and the cure of sick domesticated animals. We cannot say whether the promoters of this venture propose to have both an in- and an out-patient department, or whether the animals seeking relief will only require the passport of sickness and destitution to ensure treatment. But, speaking seriously, we must certainly say that the title of a "Hospital for Animals" is a perversion of the use of words. The Fulham Road, we know, boasts a "Dolls' Hospital," and in a street off Shoreditch there is such an establishment as an "Umbrella Hospital." So much for the degradation and the mutability of the meaning of our words.

---

A MEN'S convalescent home is in contemplation at Llanfairfechan, in North Wales. This is a most admirable situation for such an institution, on account of its proximity alike to mountain and sea. Nowhere, perhaps, in the world is there a more delightful atmosphere or more charming scenery than at Llanfairfechan and the neighbourhood. The promoters of this movement will do well to carry out their excellent intention.

A special meeting of the Governors of the Royal South Hants Infirmary, for the purpose of electing an assistant-surgeon, in the room of Dr. Wade, resigned, was held on Monday afternoon, in the infirmary premises. Mr. Stuart Macnaghten, the chairman, presided. The Chairman proposed, Mr. Grimstone seconded, and Mr. Buchan supported the motion appointing Dr. Shettle. The proposition was carried with acclamation.

---

The bazaar and theatrical performances in connection with Sir Patrick Dun's Hospital have realised a net profit of £1,000. The sum of £500 will be lodged to the credit of the treasurers of the Fever Wing Fund, and the remainder handed over to the Board of Governors for the general purposes of the hospital.

---

The people of Fleetwood seem to be in some doubt as to whether they do or do not need a cottage hospital. Some of them favour the foundation of such an institution; others think that there is no place in the world where the sick poor are better cared for than at Fleetwood. Perhaps this is so ; but a cottage hospital is a great advantage to a small community, not merely to its poor, but to its medical practitioners, and to those who have the privilege of subscribing to its funds.

---

The annual meeting of the Governors of the Birmingham and Midland Skin and Lock Hospital was held at the institution, 103, Newhall Street. The chairman, in moving the adoption of the reports, considered that they were of a very satisfactory character, especially as regarded finance, the income having exceeded the expenditure by nearly £300. A site has been secured for a new hospital in John Bright Street, and the building fund now amounts to £4,000, or about two-thirds of the sum required for building and furnishing. No further steps are to be taken towards the erection of the new building until all, or almost all, the money is promised or in hand. Special efforts are to be made for the required amount without delay.

---

The Edinburgh Association for Incurables and Longmore Hospital, which has the Queen for its patroness, is much crippled for want of adequate means. Her Majesty visited the hospital in November last, and has since given a donation of £50 to its funds. During the year 1886 the ordinary expenditure was £3,523 7*s.*, whilst in the previous year it was £2,784 1*s.*, an increase of £739 6*s.* On the other hand, the total amount of subscriptions and donations received during the year was only £2,055 8*s.* 4*d.*, whilst in 1885 it was £2,200 2*s.* 8*d.*, a decrease of £144 14*s.* 4*d.* Notwithstanding this, in 1886 accommodation was made for 12 additional in-patients, bringing the total number of beds up to 66. 176 applications were under consideration, as against 115 in 1885. The collectors are now recommencing their disinterested labours for the current year, and the committee are most anxious for such an increased amount of support as will enable them to receive more of those applicants for whom no permanent cure can be anticipated. Patients are received from all parts of the country, even from England. It is much to be desired that this most deserving charity should receive more attention and support.

At Sunderland the infirmary is largely used by seamen, and yet out of a total of about 260 ships belonging to the harbour only eight subscribe anything to its funds. It is in contemplation to build a new wing at a cost of £9,000, and it is also proposed to clear off a debt of £3,400. With the object of raising the necessary funds, circulars have been addressed to the officers and men of the ships frequenting the harbour, and to the masters it is pointed out that if each vessel will contribute the small sum of £5 annually, over £1,000 a year would be added to the resources of the hospital. Sunderland is giving a lead which may be followed by many other seaport towns. It is probable that no class contributes so little to hospitals as the officers and crews of the marine service.

---

Caernarvon is a town of 11,000 inhabitants, but apparently without any hospital accommodation whatever. The present Mayor, Mr. Councillor J. Jones, is anxious to signalise the Jubilee year by the establishment of a cottage hospital, and with this view has sent out circulars to a number of leading inhabitants. These have elicited a very favourable response, both as to donations for the building fund and annual subscriptions. The Mayor himself offers a handsome sum by way of commencing the building fund, and also an annual subscription for five years. The good people of Caernarvon should take advantage alike of the Mayor's offer and of the year of Jubilee, and secure for themselves a much needed help in times of sickness. Here is an opportunity for a display of that loyalty which it is said is rather a minus quantity in some of the Welsh towns.

---

The Imperial Institute has received very little help from Scotland : the canny Scots preferring to help institutions of their own. Glasgow seems to have shared with the rest of Scotland the preference for local schemes. Nevertheless, a very important scheme, and one which would well commemorate the Queen's Jubilee, appears rather to hang fire in the Western metropolis. A Victoria Hospital for the south side has long been in contemplation, and has met with the approval of a large number of persons who are familiar with that portion of the city. The south side has the misfortune to be inhabited by large numbers of the poor and lower middle-class—people who can contribute very little towards any scheme of such magnitude. But that is the very kind of neighbourhood in which hospitals should be placed. If Glasgow needs another hospital—and few people will say it does not—where can it so suitably be erected as at the south side, in the very midst of those who will constantly require its aid? We fear the people of the west-end of the city, many of whom are princes in point of wealth, look coldly upon the scheme, which does not immediately concern themselves or their poor dependants—but this is neither wise nor charitable, and, as we have pointed out before in these columns, Glasgow is one city, not many, like London, and it is the imperative duty of those who have the means to come to the help of those who have not. It will not be creditable to the wealthy inhabitants if this scheme is allowed to fall to the ground.

